# Evidence in Practice – A Pilot Study Leveraging Companion Animal and Equine Health Data from Primary Care Veterinary Clinics in New Zealand

**DOI:** 10.3389/fvets.2016.00116

**Published:** 2016-12-23

**Authors:** Petra Muellner, Ulrich Muellner, M. Carolyn Gates, Trish Pearce, Christina Ahlstrom, Dan O’Neill, Dave Brodbelt, Nick John Cave

**Affiliations:** ^1^Epi-interactive Ltd., Wellington, New Zealand; ^2^Institute of Veterinary, Animal and Biomedical Sciences, Massey University, Palmerston North, New Zealand; ^3^Equine Health Association, Wellington, New Zealand; ^4^The Royal Veterinary College, Hatfield, UK

**Keywords:** surveillance, veterinary, primary care, early warning, interface design, IT, companion animal, equine

## Abstract

Veterinary practitioners have extensive knowledge of animal health from their day-to-day observations of clinical patients. There have been several recent initiatives to capture these data from electronic medical records for use in national surveillance systems and clinical research. In response, an approach to surveillance has been evolving that leverages existing computerized veterinary practice management systems to capture animal health data recorded by veterinarians. Work in the United Kingdom within the VetCompass program utilizes routinely recorded clinical data with the addition of further standardized fields. The current study describes a prototype system that was developed based on this approach. In a 4-week pilot study in New Zealand, clinical data on presentation reasons and diagnoses from a total of 344 patient consults were extracted from two veterinary clinics into a dedicated database and analyzed at the population level. New Zealand companion animal and equine veterinary practitioners were engaged to test the feasibility of this national practice-based health information and data system. Strategies to ensure continued engagement and submission of quality data by participating veterinarians were identified, as were important considerations for transitioning the pilot program to a sustainable large-scale and multi-species surveillance system that has the capacity to securely manage big data. The results further emphasized the need for a high degree of usability and smart interface design to make such a system work effectively in practice. The geospatial integration of data from multiple clinical practices into a common operating picture can be used to establish the baseline incidence of disease in New Zealand companion animal and equine populations, detect unusual trends that may indicate an emerging disease threat or welfare issue, improve the management of endemic and exotic infectious diseases, and support research activities. This pilot project is an important step toward developing a national surveillance system for companion animals and equines that moves beyond emerging infectious disease detection to provide important animal health information that can be used by a wide range of stakeholder groups, including participating veterinary practices.

## Introduction

An approach to surveillance has been evolving that utilizes computerized veterinary practice management systems to capture data from individual primary-care veterinary practices and generate population-level information on animal health and welfare (1). While the concept of using veterinary clinical data and electronic medical records for animal health surveillance is not new (2), considerable advances have recently been made to improve compliance, for example, through the development of web-based data collection systems (3). An automated approach to collecting, sharing, and analyzing veterinary primary care data to understand disorders and improve the welfare of animals was initially pioneered for companion animals by the Royal Veterinary College in the UK (VetCompass^1^) and similar systems or adaptations are now emerging in different institutions and countries (1, 4, 5). The advantage of this approach is that it can be fully integrated in the veterinary workflow and captures information directly from the practice management software, thus minimizing time-consuming additional data entry and the use of linked software or websites. However, only recently resources have become available to process such large volumes of data in real-time (6, 7).

The high value of primary care data for companion animal health research has already been demonstrated. Results from several studies investigating the prevalence of common disorders and associated risk factors in dogs have filled previous knowledge gaps and identified strategies for improving canine health (8–10). Ongoing data collection will provide sufficient big data to identify additional associations and risk factors; indeed, the UK VetCompass database has collected data on more than 6 million unique animals in over 470 participating practices (11). Clinical decision-making and identification of research priorities can also be supported by the system, such as ranking of differential diagnoses, vaccine recommendations, directing veterinary education and training, and investigating changes in disease prevalence (12, 13).

However, the use of such data is by no means restricted to research and clinical decision-making and, instead, potential applications are manifold. For example, systematically collected temporal and spatial data can also be used for surveillance activities (14) and to aid decision-makers in having a sound understanding of disease prevalence, risk factors, and outcomes. A formal assessment recently provided proof-of-concept for the use of electronic medical record data to detect aberrant animal health events in space and time (15). Furthermore, both VetCompass and a similar system also developed in the UK called small animal veterinary surveillance network (SAVSNET) have been identified as mechanisms to collect antimicrobial usage and resistance data (16–18).

Importantly, the success of such an approach to animal data collection hinges on veterinary practitioners providing high quality, standardized data and, in the case of VetCompass, this has been supported by the availability of standardized diagnostic terms through the VeNom project (19). The central database dictionary developed by the VeNom Coding Group includes a standard set of clinical veterinary terms and VeNom codes for species, breeds, diagnoses, presenting complaints, procedures, and administrative tasks associated with canine, feline, and other small companion animal veterinary work. A VeNom code list for equines is also currently under development, which provides an exciting opportunity to extend the approach beyond small companion animals. A formal study is currently underway to collect critical baseline information regarding the clinic demographics. Details of veterinary practices employing registered veterinarians are formally published by the New Zealand Veterinary Council,^2^ and most are believed to work with an electronic veterinary recording system.

Despite the high utility of a VetCompass-like system, no such system currently exists on a large scale in New Zealand. However, New Zealand’s geographic isolation and unique ecosystem (20) and absence of many diseases endemic in other countries underscore the importance of gathering New Zealand-specific data on companion animals and equines to preserve the high health status of its animal population and the continued thriving of its veterinary services. In this pilot study, we explored the feasibility of a multi-species New Zealand veterinary practice-based health information and data system modeled upon the existing VetCompass system. Our main objectives were to gage end-user support, prototype the approach in the New Zealand veterinary landscape, and create preliminary system outputs prior to seeking funding for a nationwide roll-out. A special focus of the project was to work closely with participating veterinarians to gain an understanding of how the system could best be embedded in their clinical workflow.

## Materials and Methods

### Development of Software Patch

**Figure 1:**
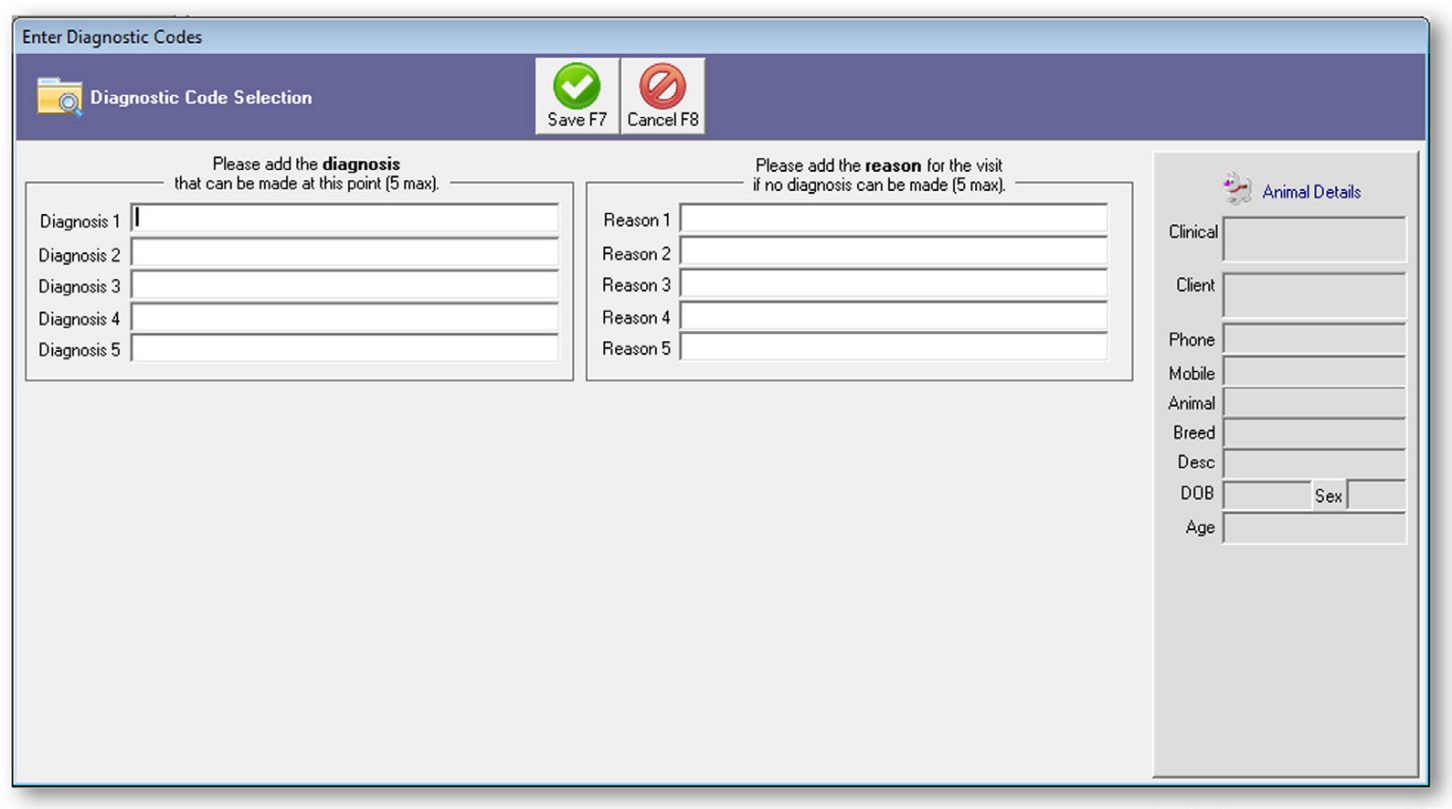
**Screenshot of code-entry for diagnosis and reason for the visit installed in the practice management software Vision PM to support the 2016 New Zealand surveillance pilot study**.

The project team developed a software add-on in collaboration with Provet Animal Health Practice Solutions (the vendor of Vision VPM), which enabled veterinarians to enter diagnoses and/or reasons for the visit directly into the practice management system. The new code-entry fields (Figure 1) were added in the workflow at a stage where the veterinarian enters and saves a visit or case, with an option to opt-out if they did not want to enter a code at this stage. Up to five diagnoses and five reasons for visit could be entered for each episode of clinical care.

The software patch was populated with VeNom codes, a suggested standard of language and terms modeled into parent-child hierarchies, that are used internationally in veterinary diagnosis and reports (19) compiled for companion animals (v20 04DEC2012) and equines (pre-release draft version). The number of diagnostic and reason for visit codes in the companion animal list was 2,322 and 1,670, while the number of codes in the equine list was 1,948 and 203, respectively. All terms were available for both series of five boxes. Companion animals were differentiated from equines in this study, as New Zealand has a large population of racehorses. In New Zealand, small animal species kept as pets (e.g., cats and dogs) are commonly referred to as companion animals, whereas horses are referred to as equines.

As the VeNom code list contained more than 6,000 single entries to choose from, a key aspect was the development of an easy-to-use user interface to search and find relevant codes. A “Google-type” autosuggest search (21) allowed users to type keywords or word fragments that filtered the list to the relevant term. Other relevant data fields to be extracted, such as species, breed, age or sex, were already captured by the standard functionality of the existing practice management system.

**Figure 2:**
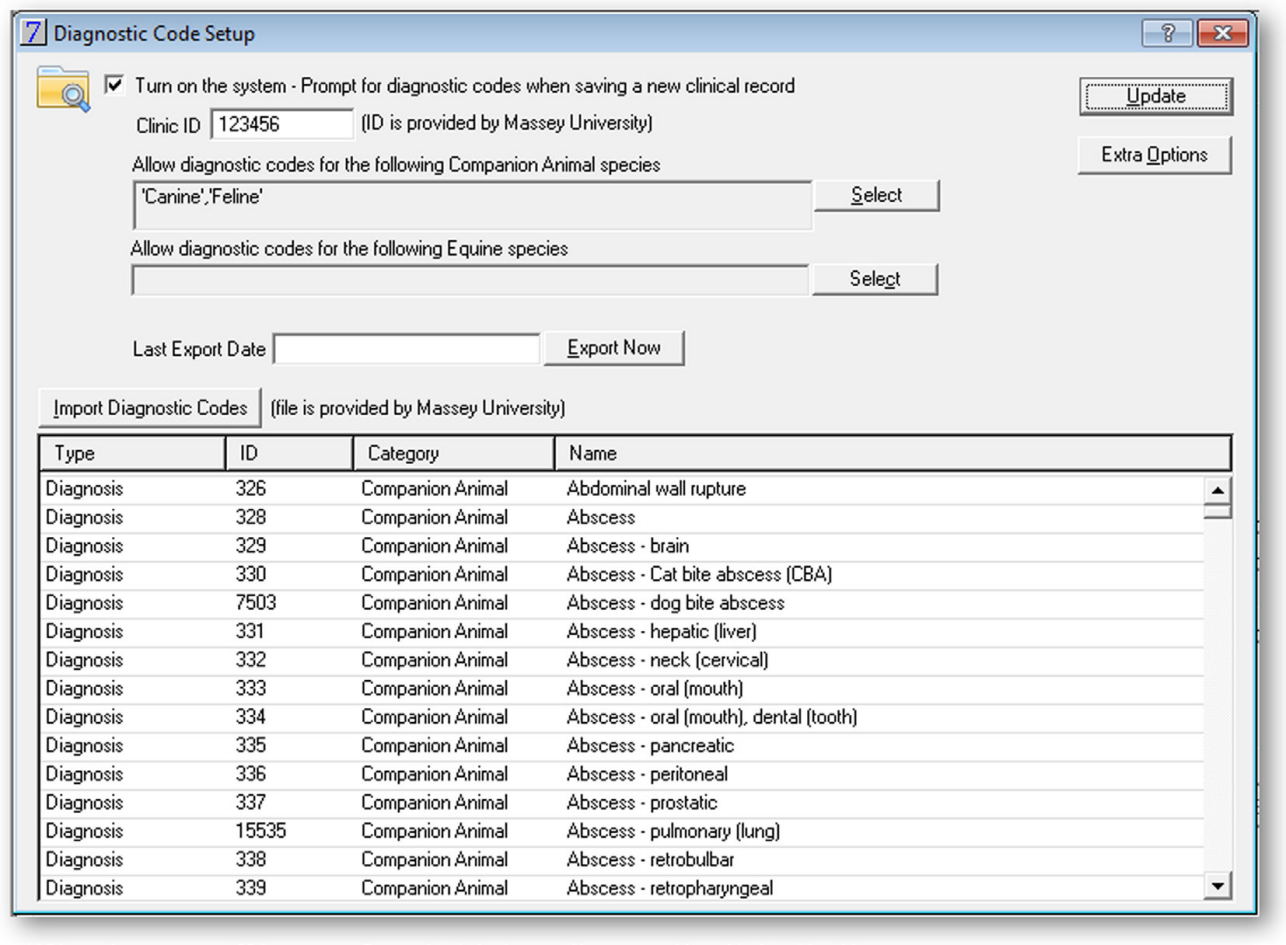
**Screenshot of the administration interface in the practice management software Vision PM to support the 2016 New Zealand surveillance pilot study**.

In addition to the VeNom term entry field, further data were collected and extraction was automated (Figure 2). This utility was password-protected and included the following features:

Opt-in for the coding.
Assignment of Clinic ID (a unique code for a veterinary practice).
Selection of the species and records to be included for the term entry and data extraction.
Ability to manually bulk export data. This function was used at the start of the pilot to extract historic data.
Diagnostic code import. This function was used to upload the VeNom coding lists and could be used to update the list at a future date.
Details of the external location where the data extract is sent to; this was set up with the details of the FTP server at Massey University.
Fields to select time of the day and frequency of the automatic data extract.

Once the data export was set up, the case information was extracted at the specified time of the day into an XML file that was sent *via* FTP to the secure servers at Massey University. A safe-guard was implemented such that the timestamp of the last successful extract was logged and data were extracted back to the time when the last extract ran successfully. This ensured that data were sent even if an export failed on a particular day.

### Practice Recruitment and Patch Installation

Two veterinary practices were recruited to participate in the 4-week pilot study, based on convenience sampling. Veterinary Clinic A was a mixed animal practice with seven veterinarians that saw both companion animal and equine patients. Veterinary Clinic B was an exclusively companion animal practice that employed two veterinarians.

Each practice was visited by a project team member to install the software patch, which was developed in collaboration with the practice management system provider, and to introduce the participating veterinarians to the system. At the time of installation, all historical records were extracted from the clinic database to provide baseline demographics and statistics on participating clinic patients. Veterinarians were briefed in VeNom codes prior to the trial and were asked to add at least one diagnosis and/or reason for visit for every patient visit. On completion of the first two weeks of the trial, preliminary results were presented to the veterinarians and their feedback was discussed.

### Information Technology and Data Extraction

**Figure 3:**
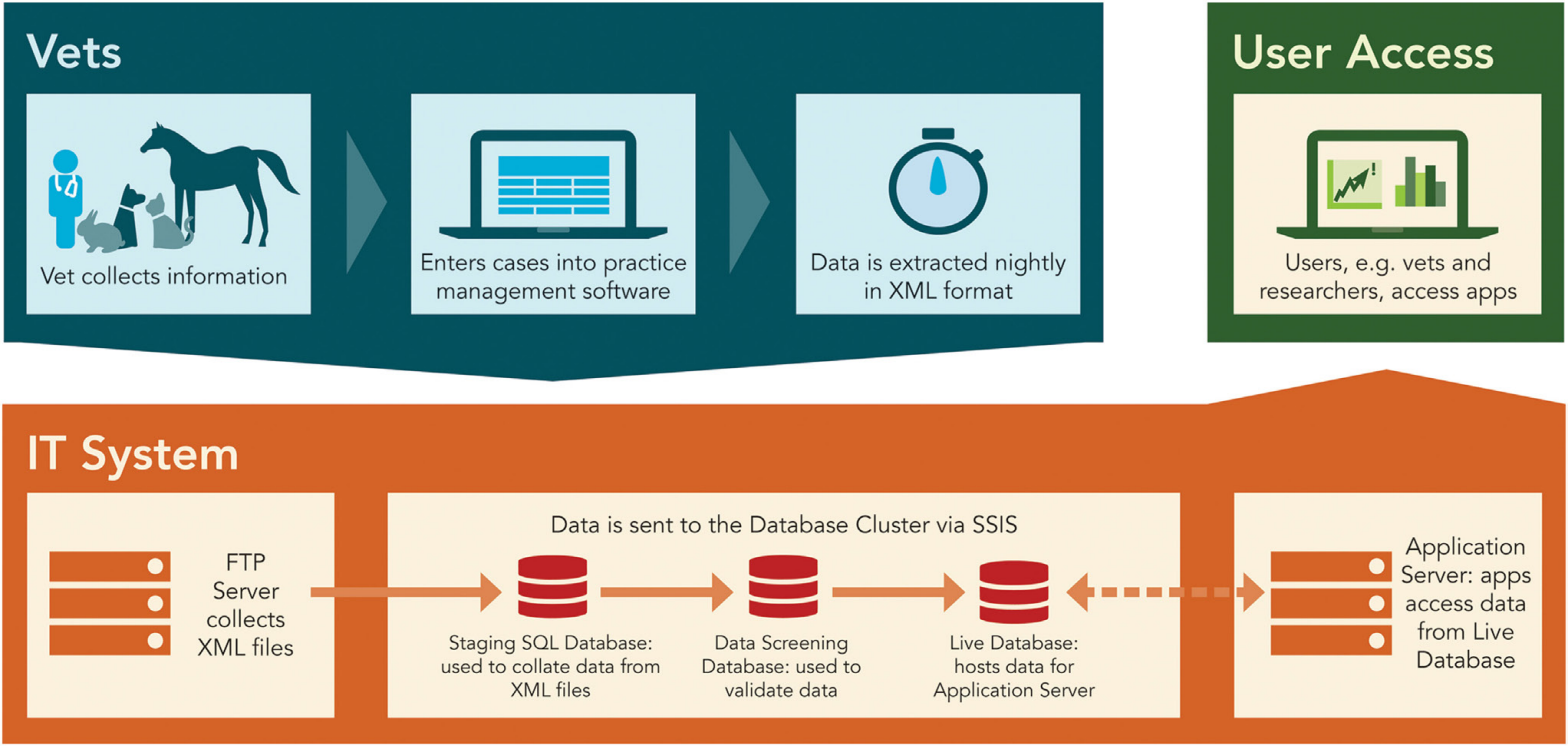
**The overall scheme and IT architecture of the data management system**.

An IT server system hosted by Massey University was set up to collect and store data from the veterinary practices. The system consisted of an FTP server to receive the XML files, an application server, as well as a shared database server (Figure 3). For development purposes, a dedicated development server was set up for testing applications and processes before implementing them in production. Data were anonymized by not extracting owner names and reducing the owner addresses to the post code only.

To process the XML files, a structured query language (SQL) Server Integration Services job was set up that migrated the data from the XML files into the “Staging” SQL server database. This database was used for data collection. Although not yet functional at the time of writing the current paper, a second database for data screening and a third database that uses the schema of the VetCompass system, to allow for international data comparison, were created to enable further development and expansion of the system at a later date. Nightly automated data extractions, which used a scheduled data push process from the practice to the system server, were set up for both veterinary clinics. The following fields were extracted:

Date
Postal code (of owner)
Practice ID
Animal unique ID
Species
Breed
Date of birth
Sex
Neuter status
Body weight (kg)
Body condition score (BCS) (1–9)
Messages and clinical notes added by the veterinarian in free text
VeNom diagnoses
VeNom presentation reason

The collection and use of the data during the pilot complied with the New Zealand Privacy Act 1993 and the Veterinary Council of New Zealand Code of Ethics under the Veterinarian Act 2005.

### Data Analysis

The nightly data extracts were aggregated and imported into R statistical software for analysis (22). Each variable was checked for coding errors, and a data dictionary was developed to map variations in terminology to describe the patient sex, breed, and neuter status within and between practices into a single unified coding system. Detailed summaries were prepared for and emailed to each participating practice and included descriptive statistics on the daily caseloads, patient demographics, reasons for presentation, and diagnoses.

## Results

From 18 September 2015 to 15 October 2015, participating veterinarians from Veterinary Clinic A recorded 120 visits for 86 unique equine patients and 241 visits for 210 unique companion animal patients, whereas participating veterinarians from Veterinary Clinic B recorded 224 visits for 168 companion animal patients. The average VeNom coding rates (i.e., visits with at least one diagnosis or reason for visit coded) were 83% for Veterinary Clinic A equine visits, 87% for Veterinary Clinic A companion animal visits, and 74% for Veterinary Clinic B companion animal visits. The average number of daily records totaled across the clinics for the 27-day pilot trial was approximately 21.

Data from the remaining fields required extensive cleaning in preparation for analysis to correct spelling errors and ensure consistent use of terminology. Equine visits were more likely to be missing information in the core data fields than companion animal visits (Table 1). The placement of the coding field was very well received by the participating veterinarians, and only minor changes were requested, for example, functionality to close the window by a single button action.

**Table 1:** **Percentage of patient visits with complete information in key data fields**.

	Veterinary Clinic A			Veterinary Clinic B	
	Feline	Canine	Equine	Feline	Canine
Client post code	94.1	93.8	90.6	92.6	91.9
Breed	100	100	84.1	100	100
Birth date	95.8	97.4	88.3	99.7	99.58
Sex	96.3	98.2	81.4	99.9	99.66
Neutered	100	100	100	100	100
Weight	89.8	96.5	0.4	98.6	99.1
Body condition score	0.4	0.1	0.1	95.7	95.9

Across both veterinary clinics, the most common reasons for cats to present were for vaccination (19%), gastrointestinal problems (11%), and non-specific clinical signs (anorexia, lethargy, and weight loss) (9%). For dogs, the most common reasons were vaccination (20%), skin disease (11%), and lameness (5%). Most Veterinary Clinic A equine cases were seen for wellness visits (17%), lameness (8%), or therapeutic interventions (3%). Over 100 unique diagnostic codes were utilized during the pilot trial.

### Usability Assessment

The practice-specific summaries prepared for and discussed with the participating veterinarians were enthusiastically received by the veterinarians. Care was taken to provide metrics not only of value to population health analyses but also to assist the veterinarians in improving their practice management. These included maps showing the distribution of clients by postal code, caseload statistics by weekday, and detailed analyses of common reasons for presentation and diagnoses. It was particularly interesting to note the number of animals that presented for routine wellness visits, but were diagnosed with other medical conditions, which highlights the importance of preventative veterinary care. Participating veterinary practices continued to code beyond the pilot period even though the data collection process was not actively monitored during this time. Coding rates remained high and informal feedback to the practices continued (data not shown).

The data migration worked seamlessly over the 4-week window, and there were no issues with transferring the nightly uploads to the server. Feedback from the participating veterinarians indicated that the VeNom codes could be improved to remove redundancies. Though they could readily work with the VeNom codes, in some circumstances they expressed uncertainty as to the best suited code(s) and noted some omissions, particularly some that are parochial or idiosyncratic to New Zealand, such as trace element deficiencies and toxicities. Otherwise, they found the search format to be user friendly and easy to operate.

## Discussion

This study provided proof-of-concept for the feasibility of establishing a VetCompass-like system for collecting veterinary clinical data on small animals and equines in New Zealand. The observed high percentage of coded cases demonstrates that veterinarians are not just willing to code clinical cases according to a set of standardized terms, but if properly engaged they show considerable enthusiasm to improve the quality and usability of the data they collect. In this study, this enthusiasm was driven by a sustainable information feedback loop providing veterinarians with easily accessible “practice health” metrics that could potentially help them to improve their clinical decision-making and business strategy (e.g., by understanding daily caseloads, patient demographics, temporal trends, and frequency of specific disorders/procedures). This, in addition to seamless integration into the existing management system and workflow, resulted in the veterinarians continuing to participate and code visits well beyond the conclusion of the pilot trial.

In human health, classification of diseases and other health problems is already standardized (23) with the International Classification of Disease published by the World Health Organization. This provides a set of agreed upon diagnostic codes (24), enabling national, regional, and global comparisons. Further development of the VeNom codes can equally enable such international collaboration in the field of veterinary medicine. As such, additional refinement and adoption of the VeNom codes to New Zealand and other countries will establish an analogous comprehensive and robust international language system for global comparisons. Furthermore, long-term national disease baseline establishment would support local comparisons, such as benchmarking to gage individual practice disease rates against a national standard.

Rapid and intuitive selection of the desired diagnosis and/or reason for visit codes by the vets within the practice management system is essential if high compliance for clinical data is to be achieved. To support this, participating veterinarians were consulted regarding the positioning of the coding in the system (i.e., between the clinical record and billing fields) to optimize workflow. Previous studies relied to varying extents on assigning codes retrospectively by processing the clinical notes added by the veterinarian either manually (25, 26) or *via* natural language processing (27). Given the remarkably high coding compliance in this study, data mining of the clinical notes could be implemented to compare and potentially validate these two different approaches and assess the accuracy of retrospectively coded cases based on clinical notes versus real-time coding at the time of the consult. The autosuggest search method for diagnostic codes and presentation reason incorporated into the New Zealand pilot system reduced the effort required by veterinarians; rather than having to scroll through long lists or decision-trees of diagnostic codes and presentation reasons, they could simply use a Google-like autosuggest function. This was well-received by all participating veterinarians and was easy to implement during development of the software patch. Additionally, the automatic daily upload of clinical data eliminated a further obstacle to compliance, as veterinarians were not tasked with manually extracting and sending data. Still, the adoption of new informatics tools depends on their usability and integration into routine workflow (28, 29), and challenges remain in convincing some veterinarians to modify existing routines and systematically record data in a usable way.

Primary care animal health data have successfully been used to estimate disorder prevalence in dogs (10), and recent work has clearly demonstrated the ability of such systems to produce reliable disease outbreak alerts (15). At the same time, this information can also provide easy and immediate access to epidemiological information relevant to a full investigation and assessment, as effective management or eradication of a new disease is often only technically feasible and affordable if emerging threats are identified early. Veterinary practitioners are often the first trained professionals to observe clinical signs in affected animals and therefore play an important role in early disease detection (30). The development of standardized and easy-to-use systems for recording clinical observation data in the field would remove a significant barrier to implementing veterinary practitioner-based disease surveillance in real-time (31–33). This would be an improvement over current systems that rely on manual curation, periodic reporting, and retrospective recording of diagnoses based on clinical notes. With the increasing sophistication of IT systems, such big data can now be securely collected, analyzed, and presented automatically.

The primary care data collection described here can easily form part of national animal health surveillance activities by collecting data on specific disorders diagnosed (4, 34–36). As such, we believe this new approach to surveillance should not be classified as being “syndromic” in nature (37) but rather be perceived as access to a new type of (big) data. To date, such big data on companion animal health have not been immediately available and had to be, with considerable effort, collected outside of the primary veterinary care environment (e.g., through data entry on dedicated websites) (33, 38). However, the overall system design and the nature of the data extracted will ultimately define the characteristics of the surveillance conducted (39).

This pilot demonstrated high feasibility for primary care data collection and surveillance in different species groups of animals under veterinary care (i.e., companion animals and equines), and the system could in theory be extended to every species for which electronic primary care data exist. It extends previous companion animal focused work to also include equine, which includes not only horses held for recreational use but also for racing and other sporting purposes. As a result, the project was supported by the New Zealand equine industry that has a strong international reputation for producing high-quality performance horses and breeding stock (40). A similar extension to livestock is possible with appropriately developed VeNom-like codes and a customized data entry interface. This could expand early warning capacity and support biosecurity preparedness and government-industry collaboration in the livestock sector. It could also facilitate trade by providing assurance to trade partners using baseline information on the number of animals seen by a veterinarian and thereby demonstrating evidence for sufficient coverage of care by veterinary services. Furthermore, such a system could be adapted to monitor antimicrobial usage (16) and resistance (AMR) in companion animals and livestock; hence, the value of the piloted system to AMR monitoring should be further explored along with necessary requirements.

As this was a pilot study, there were some limitations. Available funding was only sufficient to enroll two practices and to work with one software provider to build the modifications required to extract the data and enter the coding field software. However, many additional software providers as well as veterinary clinics expressed interest in participating, which has supported the further development of this pilot system. An additional limitation was that the equine VeNom codes were only a draft version compiled by the RVC and were not yet publically released at the time of data collection. Furthermore, we observed that the BCS coding field was coded at a lower rate than other variables in Clinic A. Going forward, the epidemiological value and validity of this (and other coding fields) will have to be formally assessed to define in more detail how the information extracted can be best utilized. Furthermore, such disparities in coding rates between clinics may encourage improved coding if these data are communicated back to the clinics. As with all analyses based on big data, inherent biases stemming from multi-source data collection must be considered (7).

Ideally, any practitioner-based disease surveillance system would be supported by a multi-tiered user interface where veterinary clinics could access their individual practice information and benchmark their metrics to national/regional levels. It is believed that tangible and even monetizable benefits (such as practice health summaries, evidence for clinical decision-support, or performance indicators) are required for the participating practitioners to comply fully and that without these benefits larger national or international research objectives are insufficient to motivate participation. A customized suite of data analysis and visualization tools are currently being developed that allows users to easily explore animal health trends while maintaining the confidentiality of animal owners and their veterinarians. Indeed, sophisticated web-based applications are increasingly being used for surveillance and complex epidemiological data analysis/visualization (28). For example, a prototype of an animal health and slaughterhouse surveillance data visualization system was recently developed that demonstrated the value of an interactive data hub for the early detection of outbreaks and health trends for equine surveillance in Switzerland (3, 33). The SAVSNET in the United Kingdom has recently started publishing quarterly reports (4) on national practice- and laboratory-based surveillance, though the presentation reasons and diagnoses collected are not based on standardized terms. By integrating knowledge and experience of multiple clinical practices into a common operating picture, it would be possible to observe high-level trends that are valuable for not only veterinarians but also researchers and decision-makers as well. This, supplemented with coding at the time of consult, would facilitate timely communication, as data would be available near real-time and analyzed automatically.

Further work should consider an enrollment strategy for a nationwide surveillance system, such as whether all New Zealand veterinary clinics should be included or only selected “sentinel” practices. Given the number of registered veterinary practices in New Zealand and that the average number of daily records recorded during the pilot was 21, we could conservatively expect at least 3,000 records per day coming into the system. Insights from the analysis of surveillance reports and participant feedback highlight important considerations for transitioning the pilot program to a sustainable large-scale and multi-species surveillance system. Other veterinary practitioner-based surveillance systems have used financial incentives such as laboratory testing credits (32) and direct financial compensation (41) to encourage user participation. Although this has been highlighted as an important incentive for some veterinarians (31), it may not be sustainable depending on long-term funding availability. The strong support from veterinary clinics in the current study suggests alternative engagement strategies may be more effective, as intrinsic motivation (e.g., to understand the clinic’s metrics and how it compares to regional statistics) may be a stronger driver for increased participation. As the system develops, it will be important to investigate additional practice management software programs, their market share in New Zealand and how they differ to Vision VPM used in this pilot.

In conclusion, recent advances in technology have made it possible to integrate large volumes of clinical data from practicing veterinarians into population health data in near real-time. This provides new opportunities in many areas including detection of unusual health events (36), provision of benchmark information, and provision of information on breed health (42). The high usability demonstrated in this New Zealand pilot study has provided a first step in implementing sustainable surveillance based around primary care data. Without adding undue burdens on participating veterinarians and veterinary clinics, this system captured information on presentation reason and subsequent diagnoses. By embedding into the existing workflow, we have created an interface that allows extraction of information from veterinarians that was previously poorly accessible. The set-up creates a win-win for veterinary practices, population health, and individual animal evidence-based medicine. The insights gained from these data will be used to refine the surveillance system design for implementation on a national scale, and a strategy for rollout to practices across the country is currently being developed.

## Author Contributions

PM, CA, and UM drafted the manuscript and made a lead contribution to the conception, design, analysis, and interpretation of the work. NC and TP critically reviewed the paper for important intellectual content and made a lead contribution to the conception, design, analysis, and interpretation of the work. MG contributed to drafting and reviewed the manuscript and made contributions to analysis and interpretation of the work. DO and DB contributed to the conception and design of the work and reviewed the manuscript.

## Conflict of Interest Statement

The authors declare that the research was conducted in the absence of any commercial or financial relationships that could be construed as a potential conflict of interest.

## Footnotes

^1^www.rvc.ac.uk/VetCompass.

^2^http://www.vetcouncil.org.nz/documentation/VetRegister/VetRegister_Practices.pdf.
