# Corrigendum: Evidence in Practice – a Pilot Study Leveraging Companion Animal and Equine Health Data From Primary Care Veterinary Clinics in New Zealand

**DOI:** 10.3389/fvets.2021.789718

**Published:** 2021-11-01

**Authors:** Petra Muellner, Ulrich Muellner, M. Carolyn Gates, Trish Pearce, Christina Ahlstrom, Dan O'Neill, Dave Brodbelt, Nick John Cave

**Affiliations:** ^1^Epi-interactive Ltd., Wellington, New Zealand; ^2^Institute of Veterinary, Animal and Biomedical Sciences, Massey University, Palmerston North, New Zealand; ^3^Equine Health Association, Wellington, New Zealand; ^4^The Royal Veterinary College, Hatfield, United Kingdom

**Keywords:** surveillance, veterinary, primary care, early warning, interface design, IT, companion animal, equine

In the original article, the **Conflict of Interest** Statement was incomplete. The corrected statement appears below.

“The Handling Editor AP declared a past co-authorship with the author PM and states that the process nevertheless met the standards of a fair and objective review.

The remaining authors declare that the research was conducted in the absence of any commercial or financial relationships that could be construed as a potential conflict of interest.”

The authors apologize for this error and state that this does not change the scientific conclusions of the article in any way.

## Publisher's Note

All claims expressed in this article are solely those of the authors and do not necessarily represent those of their affiliated organizations, or those of the publisher, the editors and the reviewers. Any product that may be evaluated in this article, or claim that may be made by its manufacturer, is not guaranteed or endorsed by the publisher.

